# Emergence of single-molecular chirality from achiral reactants

**DOI:** 10.1038/ncomms6543

**Published:** 2014-11-21

**Authors:** René R. E. Steendam, Jorge M. M. Verkade, Tim J. B. van Benthem, Hugo Meekes, Willem J. P. van Enckevort, Jan Raap, Floris P. J. T. Rutjes, Elias Vlieg

**Affiliations:** 1 Institute for Molecules and Materials, Radboud University Nijmegen, Heyendaalseweg 135, 6525 AJ Nijmegen, The Netherlands; 2 Leiden Institute of Chemistry, Leiden University, Einsteinweg 55, 2333 CC Leiden, The Netherlands

## Abstract

The synthesis of enantiopure molecules from achiral precursors without the need for pre-existing chirality is a major challenge associated with the origin of life. We here show that an enantiopure product can be obtained from achiral starting materials in a single organic reaction. An essential characteristic of this reaction is that the chiral product precipitates from the solution, introducing a crystal–solution interface which functions as an asymmetric autocatalytic system that provides sufficient chiral amplification to reach an enantiopure end state. This approach not only provides more insight into the origin of life but also offers a pathway to acquire enantiopure compounds for industrial applications.

## Abstract (web summary)

Conversion of achiral starting materials into enantiopure products without additional chiral additives is a challenging task. Here, the authors show a reaction where the precipitation of chiral product induces autocatalysis, ultimately leading to an enantiopure compound.

## Graphical abstract

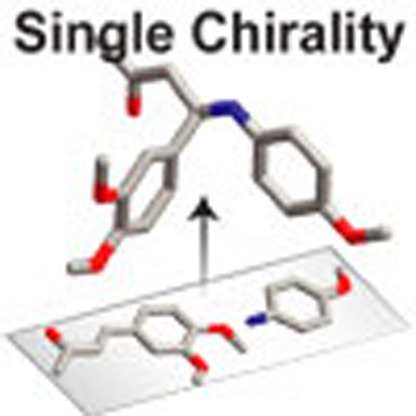

Single chirality can be considered as a signature of life, since without nature’s selection of one chiral molecule over the other our existence would be very different, if not impossible^1^,^2^. A fascinating question in science is therefore how molecular single handedness arose from an achiral abiotic world^3^. To shed light on this fundamental issue, an extensively studied topic in chemistry is the formation of single-handed (enantiopure) molecules from achiral reactants under achiral conditions^4^. Single handedness can be created, for example, through the organization of achiral molecules into enantiopure non-covalently bound architectures, such as supramolecular assemblies^5^, liquid crystals^6^ or crystals^7^. However, the synthesis of intrinsically chiral molecules of single handedness from achiral reactants still remains a major challenge. The molecular building blocks of life, for example, amino acids and sugars, as well as many pharmaceutical drugs are intrinsically chiral. The intrinsic chirality of a molecule is determined by its chiral centre and in synthesis, molecules are formed without a preference for the handedness of the chiral centre. Chiral amplification in a synthetic organic reaction is found to be extremely difficult to achieve without the help of an asymmetric catalyst.

Intrigued by this problem, Frank^8^ anticipated in 1953 that an asymmetric reaction from achiral reactants could be possible if the chiral product acts as an asymmetric catalyst for its own production (asymmetric autocatalysis). This concept of self-replication was demonstrated in solution by means of the Soai reaction^9^, which forms the landmark experiment of an asymmetric autocatalytic reaction. Typically, the Soai reaction gives the product in solution in favour of the enantiomer, which at the onset is present in the largest amount. Starting the reaction from achiral conditions results in an amplification in enantiomeric excess (*ee*) ranging from 15 to 91% (ref. 10), which can be further enhanced if the reaction product is repeatedly isolated and subjected to a new Soai reaction^11^. The necessity of this repetition emphasizes the fact that creating chiral discrimination and amplification under achiral reaction conditions in solution is a considerable challenge.

Crystal–solution interactions may be exploited to reach a stronger chiral discrimination. Chiral molecules that crystallize as a mixture of separate enantiopure single crystals (that is, racemic conglomerate crystals) are of particular interest, as was shown by the pioneering work by Havinga^12^,^13^. He discovered that an enantiomerically enriched solid state can be acquired through crystallization from a solution in which the chiral molecules can rapidly racemize through the reverse reaction. The experiments conducted by Havinga were not intended to obtain a high *ee* in high yield but instead to show that optically active compounds can spontaneously be formed. More recent studies have shown that racemic conglomerate crystals in combination with a saturated solution can be completely transformed into an enantiopure (100% *ee*) solid state by attrition-enhanced deracemization^14^,^15^. This process is named Viedma ripening with which crystals of chiral molecules can be completely deracemized^16^. It has been reported that Viedma ripening can also be applied to molecules, which racemize in solution through the reverse reaction, although in these cases a significant *ee* was required from the start to successfully increase the *ee*^17^,^18^. The powerful chiral amplification properties of crystal–solution interactions are well documented^19^,^20^,^21^; however, these conditions have never been adopted in a construction reaction^22^,^23^ to form enantiopure compounds from achiral reactants.

Here we merge such a construction reaction with Viedma ripening to overcome the weak chiral discrimination in solution-phase chemistry. This powerful combination can completely transform achiral reactants into an enantiopure solid product. Instead of asymmetric autocatalysis in solution, the results reported here show that an even stronger chiral amplification can be realized by using asymmetric autocatalytic crystal–solution interactions.

## Results

### Reaction at a low concentration

**Figure 1:**
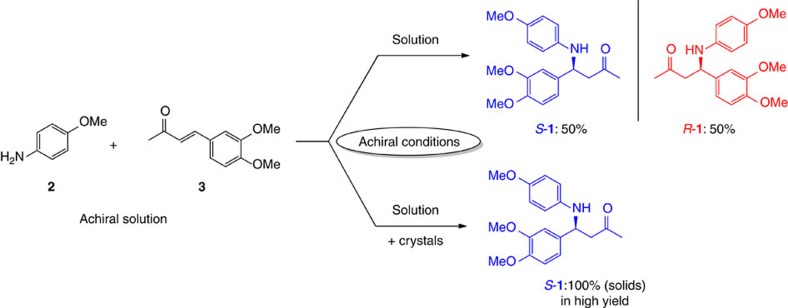
Reaction of achiral reactants 2 and 3 under achiral conditions to furnish product **1**. In solution, both product enantiomers (*R*-**1** and *S*-**1**) are obtained in equal amounts. With the combination of crystals and solution, an enantiopure solid product can be obtained.

We demonstrate this novel route to single chirality through the synthesis of the chiral amine **1** (Fig. 1). This product is formed directly in an aza-Michael reaction from the achiral reactants *p*-anisidine (**2**) and α,β-unsaturated ketone (**3**) using an achiral catalyst.

**Figure 2:**
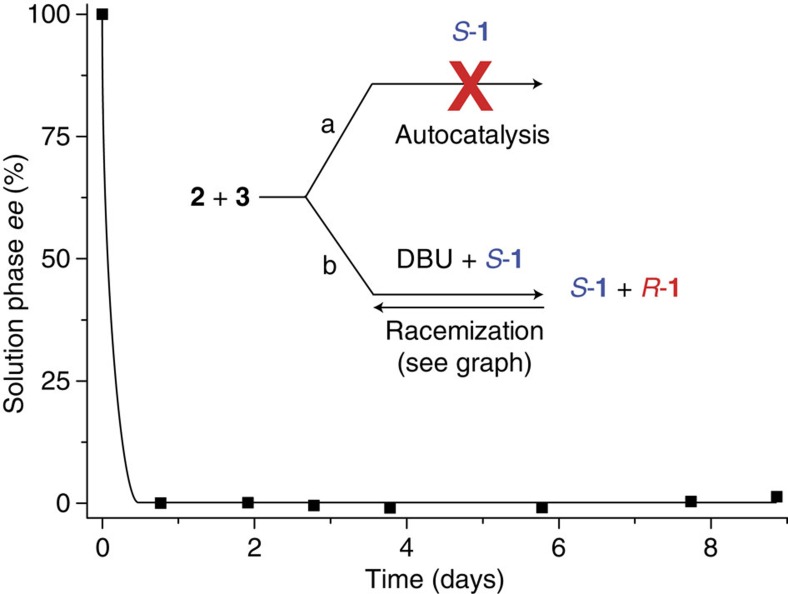
The aza-Michael reaction in a diluted solution (0.025 M). The product cannot catalyse the reaction (pathway a). It was found that DBU catalyses the reaction both ways so that the product racemizes in solution (pathway b).

In solution, it was found that 1,8-diazabicyclo[5.4.0]undec-7-ene (DBU) is a suitable catalyst for the forward aza-Michael reaction^24^ and at the same time also catalyses the retro reaction^25^ (see Supplementary Fig. 1). Therefore, product **1** racemizes in solution through the reverse reaction as opposed to a typical racemization process (deprotonation—protonation). In solution and under achiral reaction conditions, the synthesis leads to an equal amount of right- (*R*-**1**) and left-handed (*S*-**1**) versions of the product. Since Mannich bases may catalyse their own formation in solution^26^,^27^,^28^, we also attempted to catalyse the reaction asymmetrically using the enantiopure Mannich product as a catalyst (Fig. 2). However, it was found that the product is not suited to catalyse its own formation in solution. Also in the presence of DBU, the enantiopure product still did not influence the reaction asymmetrically. Instead, a racemic solution was obtained due to the reversible reaction and this shows that there is no chiral amplification in solution.

### Reaction at a high concentration

**Figure 3:**
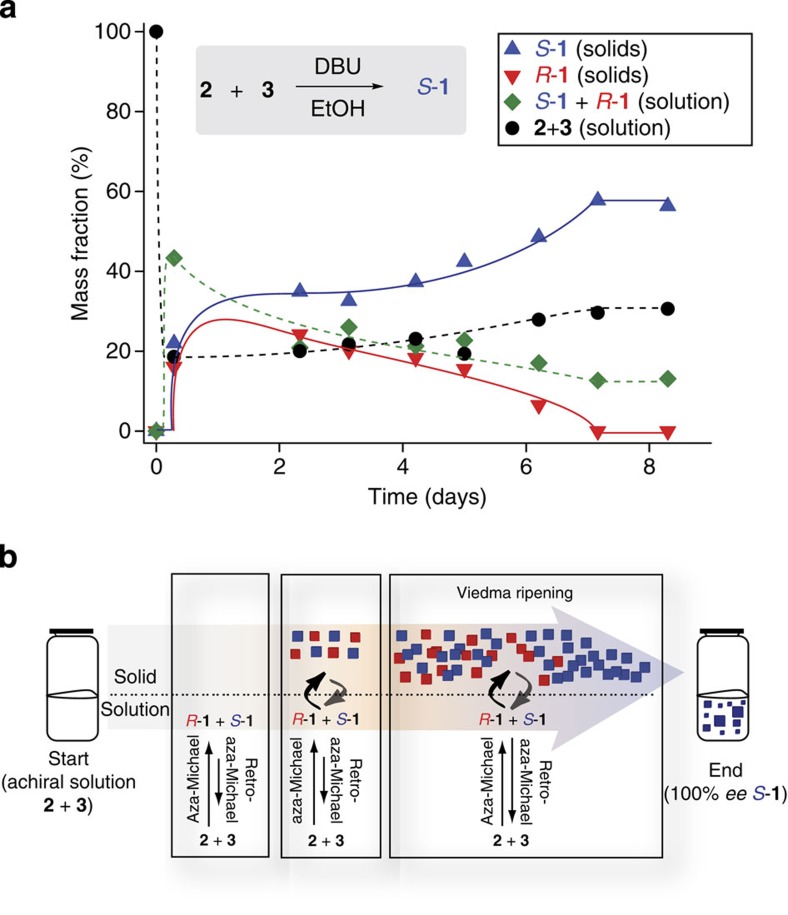
The aza-Michael reaction at a high concentration (0.5 M) in the presence of an achiral catalyst (DBU). (**a**) Evolution of the reaction in time. The lines are a guide to the eye. (**b**) Schematic representation of the mechanism behind the reaction.

To overcome the lack of chiral amplification in solution, crystal–solution interactions were utilized leading to a much stronger chiral amplification. Conducting the reaction at higher concentrations causes precipitation of the product during the reaction. This creates a crystal–solution interface that completely transforms the initial achiral reactants into an enantiopure solid end state. The course of this reaction at higher concentration is shown in Fig. 3a, while the mechanism behind the reaction is indicated in Fig. 3b.

### Mechanism behind the reaction

Once the reaction commences in solution, the achiral reactants rapidly react to give both enantiomers of the product in equal amounts because no chiral bias is present. As the reaction progresses, the solution becomes saturated with the poorly soluble product, and both enantiomers of the product precipitate in equal amounts after 0.5 days as racemic conglomerate crystals (see Supplementary Figs 2, 4 and Note 1). The initial symmetry of this solid state is broken due to either local statistical fluctuations in *ee*, a local difference in crystal size distribution between the enantiomers, or chiral impurities^29^,^30^. Subsequently, grinding of the crystals in combination with solution-phase racemization (Viedma ripening process) causes complete deracemization of the solids^16^. The yield of the solid product is ~70%.

### Chiral outcome and rate of the reaction

**Figure 4:**
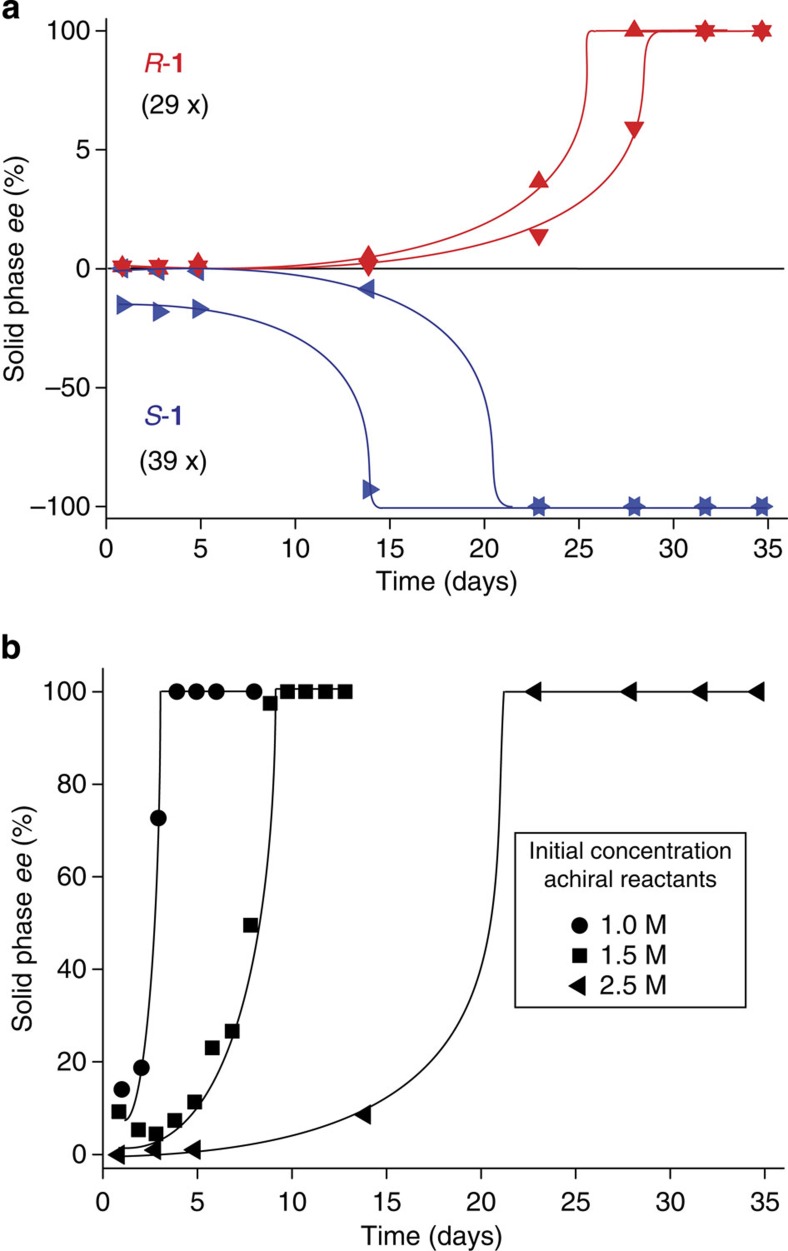
Product *ee* of the solid state against time. (**a**) Four separate experiments under identical conditions starting with an initial concentration of 2.5 M of achiral reactants. (**b**) At lower concentrations, less crystals have to be deracemized and deracemization thus proceeds faster. The lines are a guide to the eye.

The reaction leads to either enantiopure *S*-**1** or enantiopure *R*-**1** crystals. It is evident from Fig. 4a that deracemization towards *S*-**1** is faster than towards *R*-**1**. This could be attributed to traces of chiral impurities, which inhibit the crystal growth of *R*-**1** (refs 31, 32). Chiral impurities can also inhibit solution-phase processes^33^ and possibly the nucleation of the product, since in a few experiments an offset in *ee* in favour of *S*-**1** was established at the start of the precipitation (Fig. 4a). However, chiral impurities alone cannot be responsible for symmetry breaking in our experiments since deracemization also proceeds towards *R*-**1**, albeit less often. The transformation of the achiral reactants into an enantiopure product was successfully reproduced in a series of identical experiments to obtain 39 enantiopure *S*-**1** and 29 enantiopure *R*-**1** end states. Instead of using reactant **3** from a commercial source, we also used freshly prepared starting materials and again found that deracemization proceeds more often towards *S*-**1**. The enantiopure product can be obtained at an increased rate by either lowering the initial concentration of reactants (Fig. 4b) or by lowering the catalyst loading (see Supplementary Fig. 3). This, in turn, results in a lower number of crystals which have to undergo deracemization^34^. As a result, complete transformation of the achiral reactants into an enantiopure product can be realized within 3 days.

## Discussion

We have demonstrated that by combining a reversible organic reaction with Viedma ripening in the presence of an achiral catalyst, an enantiopure compound can be synthesized from achiral starting materials. Chiral amplification during a reaction can be realized without the need for rare asymmetric autocatalytic conditions in solution^35^. This conceptually new approach reported here is an alternative to the Soai-type solution-phase autocatalysis and shows that a much stronger asymmetric autocatalytic system can be realized through crystal–solution interactions. Considering the general principle that any organic reaction is reversible and that synthetic products usually are more complex and less soluble than their precursors, we envision that a wider range of chiral molecules is accessible in enantiopure form through this new approach. The facile isolation of the crystalline enantiopure product with high yield renders laborious work-up procedures obsolete and makes this an appealing method to obtain enantiopure pharmaceutically relevant building blocks. Moreover, in view of the achiral reaction conditions, this reaction proves that an enantiopure compound can simply emerge from an achiral abiotic setting. Precipitation-induced chiral amplification during synthesis therefore could provide a novel view on the initial stage of the primitive chemical processes, which ultimately led to the chemical foundation of life.

## Methods

### General methods

No chiral chemicals were used for the experiments. All chemicals, solvents and glass beads (*ø*=1.5–2.5 mm) were purchased from Sigma-Aldrich and used as received. Compound (*E*)-4-(3,4-dimethoxyphenyl)but-3-en-2-one (**3**) (98% pure) was acquired from Alfa Aesar and used as received. In addition, compound **3** was also prepared in our laboratories according to a literature procedure^36^ (the procedure is reported below). Scintillation flasks and polytetrafluoroethylene-coated oval magnetic stirring bars (length 20 mm, *ø*=10 mm) were purchased from VWR.

### Preparation and characterization of compound **3**

(*E*)-4-(3,4-dimethoxyphenyl)but-3-en-2-one (**3**) was prepared according to a literature procedure^36^: To a solution of veratryl aldehyde (20.4 g, 99.0 mmol) in aqueous ethanol (50:50 v/v%, 1.6 litre), acetone (43.0 ml, 585 mmol) and then a 10% aqueous solution of NaOH (144 ml) were slowly added dropwise. After 2 h, the solution was neutralized with 2 M aqueous HCl (120 ml) and the product was extracted with CH_2_Cl_2_ (3 × 150 ml). The organic phase was washed with brine (300 ml), dried (Na_2_SO_4_) and the solvent was removed under reduced pressure. The resulting oil was further purified by repeated crystallizations from Et_2_O and *n*-hexane to give enone **3** as yellow crystals (~95% pure according to H-NMR). ^1^H-NMR (300 MHz, CDCl_3_): *δ*=7.47 (d, *J*=16.2 Hz, 1H), 7.13 (dd, *J=*2.1, 8.3 Hz, 1H), 7.08 (d, *J*=2.0 Hz, 1H), 6.88 (d, *J*=8.3 Hz, 1H), 6.61 (d, *J*=16.1 Hz, 1H), 3.92 (s, 6H), 2.37 (s, 3H). ^1^H-NMR data are consistent with those reported in literature^25^.

### Sampling

A single drop of the suspension was taken from the experiment with a Pasteur pipette and was subsequently brought into an Eppendorf vial. The drop was mixed with 2-propanol (1 ml) and the resulting suspension was centrifuged at 14,000 r.p.m. for 1 min to separate the mother liquid and DBU from the solids. After centrifugation, the solution was carefully removed and the solids were used to determine the *ee*.

### Determination of *ee* by chiral HPLC analysis

To determine the *ee* using chiral HPLC, ~0.1 mg of solids was dissolved in 2-propanol (1.5 ml) in an HPLC vial. A drop of dimethyl sulphoxide was added to the HPLC sample to ensure complete dissolution of the solids. The sample was analysed using the following conditions: HPLC column Chiralpak AD-H (250 × 4.6 mm ID), injection volume 10 μl, eluent *n*-heptane/2-propanol (80/20 v/v%), flow 1 ml min^−1^, room temperature, *λ*=254 nm. Retention times: *R*-**1** 15.8 min, *S*-**1** 19.1 min, *p*-anisidine (**2**) 7.3 min, ketone (**3**) 7.3 min.

### Synthesis and characterization of **1** in the absence of crystals

A solution (0.025 M) was prepared by dissolving *p*-anisidine (**2**) (31 mg, 0.25 mmol), (*E*)-4-(3,4-dimethoxyphenyl)but-3-en-2-one (**3**) (52 mg, 0.25 mmol) and DBU (19 μl, 0.13 mmol) in EtOH (20 ml). The solution was stirred at 600 r.p.m. using an octahedral magnetic stirring bar at 22 °C. HPLC samples were taken daily to show that the *ee* of the solution remains racemic after 8 days. The mole fraction of rac-**1** was determined to be ~17% based on ^1^H-NMR analysis of the solution. ^1^H-NMR (300 MHz, CDCl_3_): *δ*=6.91–6.87 (m, 2H), 6.82–6.79 (m, 1H), 6.73–6.66 (m, 1H), 6.55–6.49 (m, 2H), 4.69 (q, *J*=6.5 Hz, 1H), 4.11 (br s, 1H), 3.85 (s, 6H), 3.70 (s, 3H), 2.89 (d, *J*=6.6 Hz, 2H), 2.11 (s, 3H). ^1^H-NMR data are consistent with those reported in literature^25^.

### Synthesis with the product as a catalyst in solution

Two solutions (0.025 M) were prepared by dissolving *p*-anisidine (**2**) (31 mg, 0.25 mmol) and (*E*)-4-(3,4-dimethoxyphenyl)but-3-en-2-one (**3**) (52 mg, 0.25 mmol) in EtOH (20 ml). *S*-**1** (21 mg, 25 mol%) and *S*-**1** (10 mg, 12.5 mol%) were added in separate flasks and both solutions were stirred at 600 r.p.m. using an octahedral magnetic stirring bar at 22 °C. HPLC samples were taken daily to show that the *ee* of the solution remains 100% *ee* in *S*-**1** after 8 days. However, the mole fraction of *S*-**1** of both experiments dropped (from 25 to 14% and from 12.5 to 10%) based on ^1^H-NMR analysis of the solution. Therefore, no evidence of asymmetric autocatalysis was observed in solution. The smaller final mole fraction can be attributed to the elimination of the product into its corresponding achiral counterparts **2** and **3**, as was observed earlier^25^.

### Synthesis with the product and DBU in solution

A solution (0.025 M) was prepared by dissolving *p*-anisidine (**2**) (31 mg, 0.25 mmol), (*E*)-4-(3,4-dimethoxyphenyl)but-3-en-2-one (**3**) (52 mg, 0.25 mmol), DBU (19 μl, 0.13 mmol) and *S*-**1** (21 mg, 25 mol%) in EtOH (20 ml). The solution was stirred at 600 r.p.m. using an octahedral magnetic stirring bar at 22 °C. HPLC samples were taken daily to show that the *ee* of the solution remains racemic after 8 days. The mole fraction of rac-**1** was determined to be ~17% based on ^1^H-NMR analysis of the solution.

### Control experiment to verify racemization in solution

A solution of enantiopure **1** was prepared by dissolving (*R*)-**1** (29.4 mg, 0.090 mmol) in ethanol (20 ml). After a sample was taken, DBU (7 μl, 0.05 mmol) was added, the solution was stirred and samples were taken every 15 min. The samples were analysed using chiral HPLC to show complete racemization of *R*-**1** within 90 min. The actual racemization rate in the grinding experiments is higher because the solution is already saturated with reactants. The results of this experiment are shown in Supplementary Fig. 1.

### General procedure to transform 2+3 into enantiopure 1

A typical experiment consists of combining the achiral reactants with the achiral catalyst in a solution in the presence of glass beads and a magnetic stirring bar. The resulting solution is subsequently stirred until solids emerge, which at the same time are ground until an enantiopure product remains. A solution of *p*-anisidine (**2**) (154 mg, 1.25 mmol), (*E*)-4-(3,4-dimethoxyphenyl)but-3-en-2-one (**3**) (258 mg, 1.25 mmol) and DBU (93 μl, 0.62 mmol) in EtOH (2.5 ml) was stirred at 800 r.p.m. using an oval magnetic stirring bar in the presence of glass beads (*ø*=1.5–2.5 mm, 7.0 g) in a sealed scintillation flask at 22 °C. After prolonged grinding, the solid phase of 4-(3,4-dimethoxyphenyl)-4-((4-methoxyphenyl)amino)butan-2-one (**1**) evolved to enantiopure solids, which were isolated by centrifugation in the pure form (291 mg, 71%).

### Experiment to determine concentration of 1, 2 and 3 during reaction

A solution of *p*-anisidine (**2**) (616 mg, 5.00 mmol), (*E*)-4-(3,4-dimethoxyphenyl)but-3-en-2-one (**3**) (1,032 mg, 5.00 mmol) and DBU (372 μl, 2.50 mmol) in EtOH (10.0 ml) was stirred at 1,400 r.p.m. using an oval magnetic stirring bar in the presence of glass beads (28.0 g) in a round bottom flask at 22 °C. The solid phase was isolated from the liquid phase through centrifugation. After all of the solvent was evaporated, the mass of the liquid and solid phase was measured. The concentration of reactants and product in the liquid sample was determined using ^1^H-NMR and the *ee* of the solids was measured using chiral HPLC.

### Amplification of *ee* as a function of catalyst concentration

Instead of using reactant **3** (98% purity) from the commercial source, we used freshly prepared reactant **3** (~95% purity) for these experiments:

To study the effect of catalyst concentration, we dissolved a fixed amount of reactants (*E*)-4-(3,4-dimethoxyphenyl)but-3-en-2-one (**3**) (644 mg, 3.12 mmol), *p*-anisidine (**2**) (385 mg, 3.12 mmol) and different amounts of DBU (140–233 μl, 0.94–1.56 mmol). The results of this experiment are shown in Supplementary Fig. 3.

## Author contributions

R.R.E.S., H.M., F.P.J.T.R. and E.V. designed the experiments and analysed the data. R.R.E.S., J.M.M.V. and T.J.B.v.B. performed the experiments. H.M., F.P.J.T.R. and E.V. directed the project. R.R.E.S. wrote the manuscript with contributions from all the authors. All authors contributed to the discussions.

## Additional information

**How to cite this article:** Steendam, R. R. E. *et al*. Emergence of single-molecular chirality from achiral reactants. *Nat. Commun.* 5:5543 doi: 10.1038/ncomms6543 (2014).

**Accession codes:** The X-ray crystallographic coordinates for compound **1** reported in this Article have been deposited at the Cambridge Crystallographic Data Centre (CCDC), under deposition number CCDC 976528. These data can be obtained free of charge from The Cambridge Crystallographic Data Centre via www.ccdc.cam.ac.uk/data_request/cif.

## Supplementary Material

Supplementary Information Supplementary Figures 1-4, Supplementary Note 1 and Supplementary Reference
