# Supplementary material for: Emergence of single-molecular chirality from achiral reactants
Source: Nat Commun. 2014 Nov 21;5:5543. doi: 10.1038/ncomms6543 (PMC4263183; doi:10.1038/ncomms6543)
Supplement: Supplementary Information — Supplementary Figures 1-4, Supplementary Note 1 and Supplementary Reference [file ncomms6543-s1.pdf]

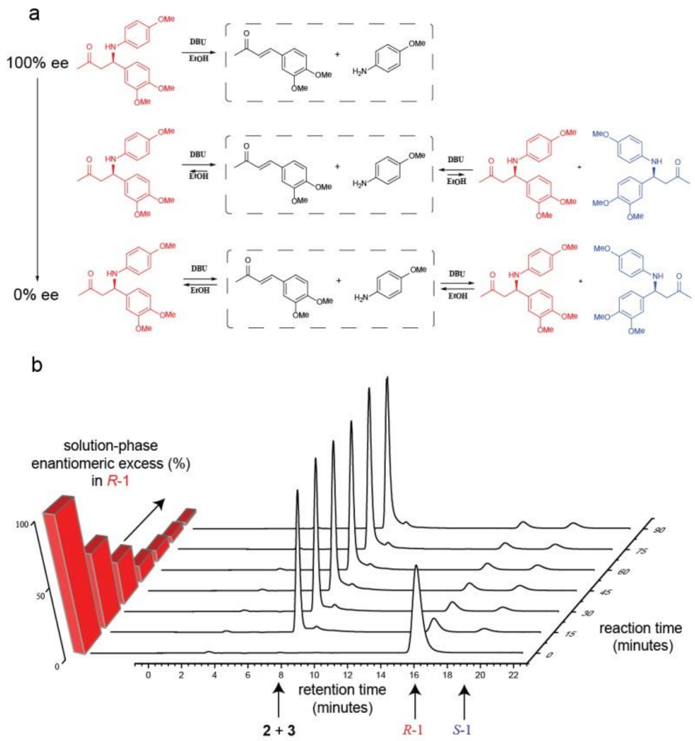

Supplementary Figure 1 | Racemization of compound 1 in solution. (**a**) The initially enantiopure *R*-**1** in solution splits up to furnish the achiral reactants **2** and **3** which subsequently react to give both *R*-**1** and *S*-**1**, thus ultimately causing the solution to become racemic. (**b**) Racemization of *R*-**1** in solution in time.The left part of the figure shows the decrease in ee of *R*-**1** as a function of time and the right part shows the corresponding HPLC traces which indicates that racemization occurs via its reactants.

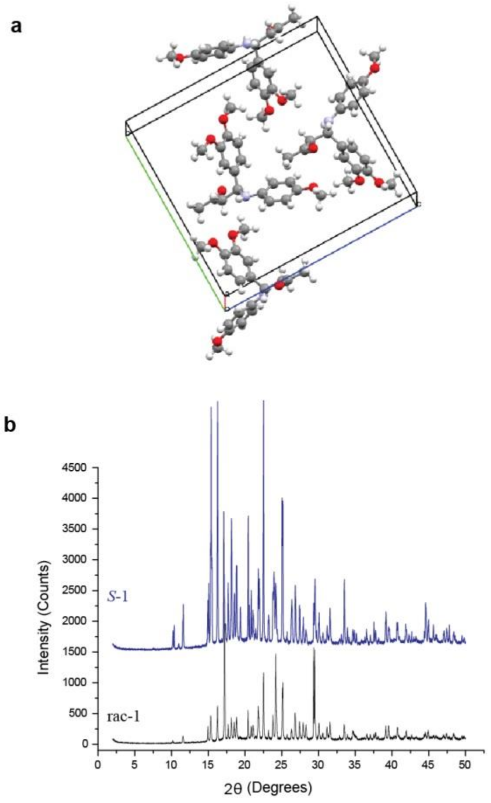

Supplementary Figure 2 | X-ray diffraction of compound 1. (**a**) The unit cell of rac-**1** which consists of four of the same enantiomers. (**b)** X-ray powder pattern of *S*-**1** (top) and rac-**1** (bottom).

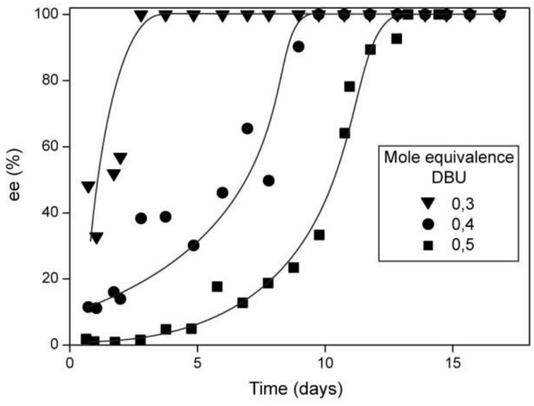

Supplementary Figure 3 | Catalyst loading. Chiral amplification as a function of time for experiments with different catalyst loadings. Lines are a guide to the eye.

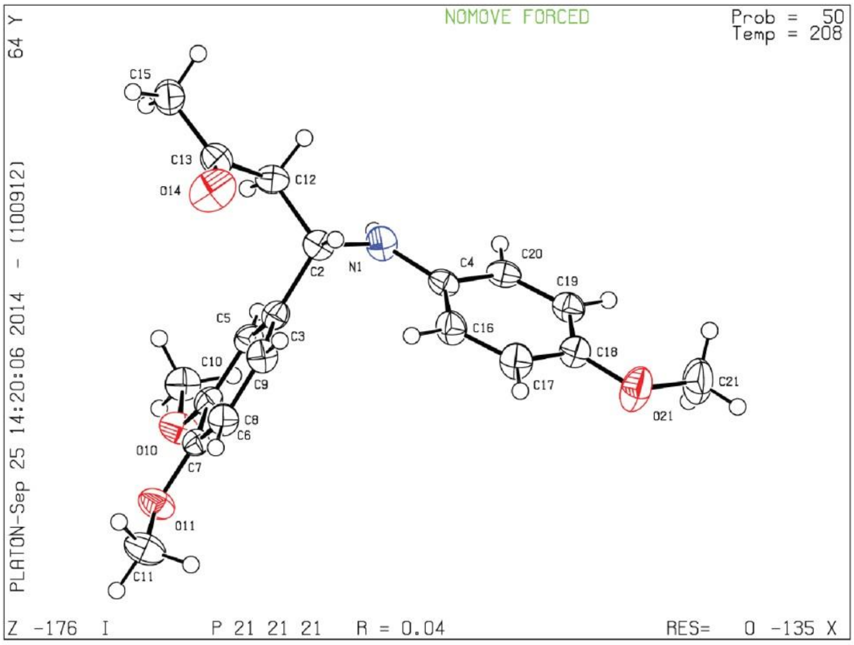

Supplementary Figure 4 | ORTEP representation of compound 1.

Supplementary Note 1

A prerequisite for chiral amplification by Viedma ripening is that the molecule must crystallize as racemic conglomerate crystals. In order to establish whether product **1** crystallizes as conglomerate crystals, X-ray diffraction studies were carried out. It was found that the unit cell of rac-**1** consists of four of the same enantiomers, proving that crystals of product **1** are indeed conglomerate crystals. In addition, if a compound crystallizes as conglomerate crystals, then the X-ray powder pattern of the racemate should match with the powder pattern of the enantiopure compound.[1] It was found that the powder patterns of rac-**1** indeed matches with the powder pattern of *S*-**1** and thus that product **1** crystallizes as conglomerate crystals. The results are shown in Supplementary Figure 2.

Crystal data of rac-**1** (recrystallized from EtOAc); $C_{19}H_{23}NO_4$, orthorhombic, space group $P2_12_12_1$, a = 6.0842 (5) Å, b = 16.1486 (8) Å, c = 17.3137 (8) Å, Z=4, Z'=1, V = 1701.09 Å^3. CCDC 976528 contains crystallographic data.

Supplementary Reference

1 Eliel, E. L. & Wilen, S. H. *Stereochemistry of Organic Compounds*. (John Wiley & Sons, Inc., 1994).
